# Towards early detection of neurodegenerative diseases: A gut feeling

**DOI:** 10.3389/fcell.2023.1087091

**Published:** 2023-02-07

**Authors:** Stephanie Makdissi, Brendon D. Parsons, Francesca Di Cara

**Affiliations:** ^1^ Dalhousie University, Department of Microbiology and Immunology, Halifax, NS, Canada; ^2^ IWK Health Centre, Department of Pediatrics, Halifax, Canada

**Keywords:** gut-brain axis, a-synuclein, A β amyloid, microbiota, parkinson disease, neurodegenerative disease, peroxisomes, alzheimer disease

## Abstract

The gastrointestinal tract communicates with the nervous system through a bidirectional network of signaling pathways called the gut-brain axis, which consists of multiple connections, including the enteric nervous system, the vagus nerve, the immune system, endocrine signals, the microbiota, and its metabolites. Alteration of communications in the gut-brain axis is emerging as an overlooked cause of neuroinflammation. Neuroinflammation is a common feature of the pathogenic mechanisms involved in various neurodegenerative diseases (NDs) that are incurable and debilitating conditions resulting in progressive degeneration and death of neurons, such as in Alzheimer and Parkinson diseases. NDs are a leading cause of global death and disability, and the incidences are expected to increase in the following decades if prevention strategies and successful treatment remain elusive. To date, the etiology of NDs is unclear due to the complexity of the mechanisms of diseases involving genetic and environmental factors, including diet and microbiota. Emerging evidence suggests that changes in diet, alteration of the microbiota, and deregulation of metabolism in the intestinal epithelium influence the inflammatory status of the neurons linked to disease insurgence and progression. This review will describe the leading players of the so-called diet-microbiota-gut-brain (DMGB) axis in the context of NDs. We will report recent findings from studies in model organisms such as rodents and fruit flies that support the role of diets, commensals, and intestinal epithelial functions as an overlooked primary regulator of brain health. We will finish discussing the pivotal role of metabolisms of cellular organelles such as mitochondria and peroxisomes in maintaining the DMGB axis and how alteration of the latter can be used as early disease makers and novel therapeutic targets.

## Introduction

Neurodegenerative diseases (NDs) such as Alzheimer disease (AD) and Parkinson disease (PD), are a rising burden to society (Aarli et al., 2006; Feigin et al., 2019; 2020; Kassebaum, 2022). The World Health Organization heeds that the rising incidence of NDs are one of the most significant public health challenges now and in the coming decades (Aarli et al., 2006; Ou et al., 2021), as long as preventative strategies and viable treatments remain elusive (Raz, 2010; Durães et al., 2018). For a detailed clinical review of the epidemiology, we refer the reader to (Erkkinen et al., 2018).

NDs manifest with multiple clinical complications and life quality constraints. They can affect the motor system, causing ataxias (Bologna and Paparella, 2020), and the cognitive system, causing dementias. However, the etiologies of NDs are unclear due to the complex genetic and environmental factors believed to underly the mechanism of disease. Multiple studies now suggest that changes in factors outside the brain, such as diet, gut microbiota (dysbiosis), metabolism, and intestinal inflammation, are linked to the risk and progression of NDs.

For example, consuming ultra-processed food enhances the risk of developing AD (Li et al., 2022), as high-fat diets are linked to the development of chronic metabolic and inflammatory diseases associated with neuroinflammation and reduced cognitive function (Cai, 2013).

The intestinal microbiota composition is sensitive to changes in diet and to the host’s metabolic and inflammatory status. Dysbiosis of commensal populations is linked to metabolic perturbations associated with psychiatric disorders such as autism spectrum disorder (ASD) (Festi et al., 2014; Pulikkan et al., 2019) and NDs such as PD and AD (Cai, 2013; Sampson et al., 2016; Zhang et al., 2018). Additionally, prolonged antibiotic use and high-fat diets are linked to commensal dysbiosis, weak intestinal-barrier function, and dysplasia of the gut epithelium. These changes correlate with elevated local and systemic inflammation and deficiencies of nutrients essential for brain health. This is because the intestinal microbiota supply essential nutrients such as vitamin B or K and their derivatives to nourish the development of the central nervous system (CNS) (Das et al., 2019; Ma et al., 2019). For example, bacterial metabolites such as short-chain fatty acids (SCFAs), including acetic acid, butyric acid, and propionic acid, are integral for learning and memory (Trinder et al., 2017) and reduction in SCFAs is associated with inflammation in Multiple Sclerosis patients and compromised neuronal function in various NDs (Ma et al., 2019).

The intestinal epithelium plays a central role in digestion and nutrient absorption, defense against pathogens, cooperation with beneficial commensals, and production of systemic endocrine signals. Recent work has shown that NDs may be seeded distally in the intestinal epithelium before disease in the brain *via* the gut-brain axis (Shannon et al., 2012; Sánchez-Ferro et al., 2015; Brudek, 2019; Chen et al., 2019; Challis et al., 2020; Mertsalmi et al., 2020; Castelli et al., 2021). Multiple studies report the accumulation of protein aggregates, hallmark pathologies of NDs such as AD and PD, appearing in enteric neurons or the gastrointestinal epithelium years before detection in the central nervous system (Braak et al., 2003; Hawkes et al., 2010; Devos et al., 2013; Chalazonitis and Rao, 2018; Kowalski and Mulak, 2019).

NDs are also characterized by abnormalities in peroxisomes and mitochondria (Nunomura et al., 2001; Reddy and Beal, 2008; Reddy 2009; Kou et al., 2011; Cipolla and Lodhi, 2017; Aparicio et al., 2022; Fedele et al., 2022; Roczkowsky et al., 2022). These organelles are central to cellular metabolism, redox stress, and immune signaling (Johannsen and Ravussin, 2009; Kou et al., 2011; Di Cara, 2020). In the intestinal epithelium, both organelles also mediate critical interactions with the gut microbial population (Di Cara et al., 2018). Thus, the consequences of aberrant peroxisome or mitochondrial function in the intestinal epithelium might represent hallmark to early onset of NDs.

**FIGURE 1:**
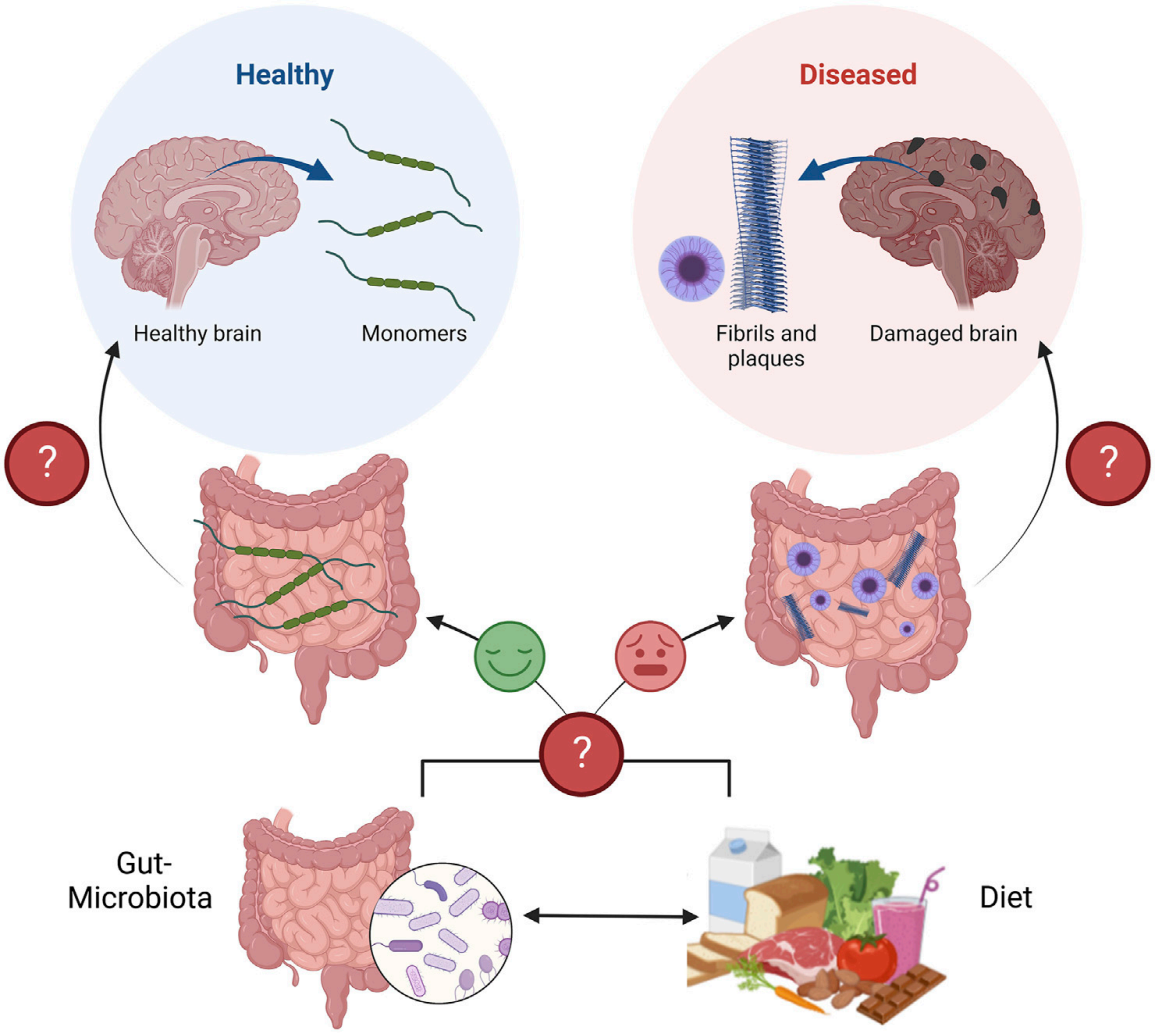
Schematic of the diet-microbiota-gut-brain axis in the context of neurodegenerative diseases. The gut, and gut-microbiota are known to secrete protective neurotrophic factors to promote brain health, neuron survival, and plasticity (Left path). If gut metabolism is perturbed, due to altered communication between the gut, and gut-microbiota, both influenced by the diet, these protective factors may not be secreted or function as intended (Right path). Perturbed gut metabolism corresponds to the appearance of neurodegenerative pathologies (Fibrils and plaques), years before appearing in the brain and causing disease. Figure constructed on BioRender.com.

Together, this evidence supports the idea that interactions between the diet, gut microbiota, and intestinal epithelium shape the lines of communication between the gut and the brain, which can protect or damage brain health (Figure 1). Understanding this communication is a promising area of research to better define the etiologies of NDs.

In this review, we discuss current evidence demonstrating that the perturbation of the host intestinal metabolism by genetics, organelle damage, or environmental factors (e.g., dietary changes or antibiotic treatment) affects the diet-microbiota-gut-brain (DMGB) signals that govern brain health representing a significant factor in the onset of NDs. We report experimental evidence demonstrating the links between alteration of the DMGB axis and NDs in studies carried out in *Drosophila* and murine model organisms. We will describe the elements of the DMGB axis in health and NDs. Finally, we will analyze the open questions in the field and discuss how unraveling the mechanisms of the DMGB axis leads to the prediction of early diagnostic markers for NDs—ultimately aiding in the development of alternative therapies that involve microbial or microbial-host metabolism targeted treatments.

## Neurodegenerative diseases

The brain is the central information processor and relay for most higher-order eukaryotes (Thau et al., 2022; Y; Wang et al., 2020). Part of the Central Nervous System (CNS), the brain is interoceptive to distal organs *via* the Peripheral Nervous System (PNS) to modulate brain-organ communication and tissue homeostasis (Buchanan and Tranel, 2009; Waxenbaum et al., 2022). In addition to direct neuronal impulses mediated by neurotransmitters, other means of crosstalk include humoral/endocrine communication by secretion of molecular agents such as hormones, lipids, and peptides (Castillo-Armengol et al., 2019); these forms of communication are bidirectional (Castillo-Armengol et al., 2019). However, cross-talk efficiency and capacity may diminish with age, as the synthesis of neurotransmitters and signaling molecules are curtailed in the brain and peripheral tissue, leading to tissue-specific/systemic diseases (Venkat et al., 2015; Ali et al., 2018). Emerging literature suggests that distal organ stress, whether metabolic or inflammatory, can impact brain function (Peters, 2006; Atamna et al., 2018).

NDs are defined by severe, progressive, and debilitating cognitive and motor disturbances (Jahn, 2013; Levenson et al., 2014; Muddapu et al., 2020) caused by neuronal death in specific vulnerable brain regions that varies with the disease such as striatal regions in PD, striatal and hippocampal and cortical regions in AD (Muddapu et al., 2020). Despite best efforts to find a cure and alleviate the Global Burden of Disease and financial burdens of NDs (Weintraub and Horn, 2008; Vossius et al., 2011; Wong, 2020; Yang et al., 2020; Ou et al., 2021), the etiologies remain elusive, and research on pathogenesis is still greatly speculative (Durães et al., 2018).

NDs are categorized into two groups; genetic and sporadic (Bekris et al., 2010; Pang et al., 2017). Sporadic NDs, contributing to over 90% of cases, are the most difficult to study because pathogenesis is driven by both spatial and temporal interactions of environmental and genetic factors, which are still largely unclear (Lu and Vogel, 2009; Pang et al., 2017; Muddapu et al., 2020; Hampel et al., 2021). On the contrary, genetic cases are much easier to study. Canonical human-linked alleles of NDs have already been identified and manipulated in many model organisms to mimic and study human disease (i.e., *Mus musculus*: mouse, and *Drosophila melanogaster*: fruit fly) (Bekris et al., 2010; Steinkamp et al., 2012). The genetic epidemiological spotlight currently points to genes that encode for proteins prone to, or that facilitate, oligomerization and aggregation (i.e., α-Synuclein and Amyloid precursor protein) or involved in autophagy and mitochondrial metabolic stress (i.e., PINK1, Parkin). The etiology of genetic NDs is also not clear due to the complexity of the mechanisms of diseases that involve both genetic and environmental factors, including diet and microbiota in both hereditary and sporadic forms (Festi et al., 2014; Hakansson, A. and Molin 2011; Hampel et al., 2021; Pang et al., 2017). Advancements in genomics, proteomics, single cell analysis, and metabolomics technology, along with multidisciplinary research approaches, have opened new frontiers in the investigations of the origins and mechanisms of disease especially sporadic NDs (Pang et al., 2017; Zheng and Chen, 2022).

The current use and repurposing of antibiotics and probiotics to treat NDs supports the hypothesis of peripheral origin of these diseases (Durães et al., 2018). This is also supported by the existence of afferent cues derived from the metabolic interactions of the microbiota, dietary factors, and the gut, which have neuroprotective effects on the brain but lead to NDs when perturbed (Brudek, 2019; Chen et al., 2019; Challis et al., 2020; Mertsalmi et al., 2020; Castelli et al., 2021). Therefore, the pathogenesis of NDs may not be confined to the brain but originate from an interorgan-communication network of the DMGB axis. Deciphering the molecular networks of the DMGB axis that distinguish between healthy individuals and patients with specific NDs represents a promising area to define not only the etiology of NDs but also the identification of early disease biomarkers to permit early intervention and prevention of disease progression.

The brain is an immune-privileged organ with a Blood Brain Barrier (BBB), a selectively permeable membrane that prevents drug diffusion into the brain (Daneman and Prat, 2015). For this reason, it is challenging to construct synthetic drugs that can easily cross the BBB to treat NDs within the brain (Pardridge, 2012) with high bioavailability in the brain tissue and without any first-pass effects (Harris et al., 2014). Therefore, alternative treatments that correct metabolic signaling at peripheral sites will alleviate the need for treatment with psychotherapeutics. Targeting the DMGB axis to address the defects that lead to disease in the gut instead of the brain offers alternative treatment options, such as dietary changes, to prevent side effects than current psychotherapeutics may cause (Winiarska-Mieczan et al., 2020). In the following sections, we will report results from studies that support the idea that the onset of NDs, such as AD and PD, begins with disturbances in the DMGB axis and that novel strategies that target this axis can address these devastating disorders.

## The gut-brain axis in alzheimer disease

### Physiopathology of alzheimer disease

Dementias, such as AD, are the most common types of NDs, with a higher prevalence in females than males (Mielke, 2018; Podcasy and Epperson, 2016; Y; Wang et al., 2020). The hallmark pathologies of AD, identified in both sexes, negatively affect cognition, predominantly long and short-term/working memory, and locomotor behavior (Clancy, 2013; Da Costa et al., 2013; Ju et al., 2013; Levenson, et al., 2014; Raskin et al., 2015; Podcasy and Epperson, 2016). The formation of aggregates of misfolded protein oligomers such as extracellular β-amyloid plaques or intracellular neurofibrillary tangles (NFTs), made of tau microtubule-associated protein (Mohandas et al., 2009; Kou et al., 2011; Hampel et al., 2021; Tracy et al., 2022) are late pathological hallmarks that cause damage to mitochondria and oxidative stress in neurons. Of protein aggregates in neurons and neuronal loss, observed as inclusion bodies that form lesions throughout the tissue (Takatori et a., 2019; Morley et al., 2018; Raskin et al., 2015; Sarantseva et al., 2009; Shadfar et al., 2015). The inclusion bodies spread over time to surrounding neurons through cell-to-cell prion-like propagation (for a detailed review on the cell-to-cell prione propagation, please refer to Hofmann et al., 2013), causing high oxidative stress and widespread inflammation of the brain, ultimately facilitating atrophy of the tissue as the disease progresses (Ashraf et al., 2014).

### Evidence of the distal seeding of alzheimer disease caused by Aβ fibrils

The Amyloid Precursor Protein (APP) is one member of a single-pass transmembrane protein family characterized by large extracellular domains. When APP is proteolytically cleaved by β/γ secretases, the product is amyloidogenic ABeta/β-amyloid peptide (Aβ) (Chen et al., 2017). The role of APP and its products is not very clear. The preeminent role of APP in the development of AD depends on the toxicity of the Aβ peptide since the loss of APP function does not seem deleterious. In AD patients, accumulation and aggregation of Aβ peptides forms Aβ fibrils that are acutely toxic to neurons. Aβ fibrils formation toxicity might explain also other pathological aspects of AD including neurofibrillary tangles, inflammation, and oxidative damage. For a detailed review on the pathogenesis of AD we refer the reader to a review by (O'Brien and Wong, 2011).

APP and β/γ secretases are not exclusive to the CNS. Recent studies reported regular APP expression in the Enteric Nervous System (ENS) of mice, suggesting an ENS involvement in AD (Van Ginneken et al., 2011; Semar et al., 2013; Chalazonitis and Rao 2018). The transgenic mice that over express a mutant form of human APP in the ENS are associated with early-onset familial AD, exhibiting an accumulation of Aβ in the enteric neurons leading to a decrease in enteric neuron abundance, dysmotility, and increased vulnerability to inflammation (Chalazonitis and Rao 2018). Preliminary data confirms that changes in location or amount of APP in the ENS correspond to disease expression in transgenic mice carrying APP mutation. APP aggregation in ENS appeared before any disease sign was detectable in the brain. Similarly, Αβ deposits have been observed in the Gastrointestinal (GI) tract of patients that overexpress APP (Semar et al., 2013; Sun et al., 2020). Although the study of the role of Aβ in the gut in the context of AD is still in its infancy, there have been increasing evidence that support a model where Aβ triggers NDs from the gut in the brain (Braak et al., 2003; Chalazonitis and Rao, 2018; Kowalski and Mulak., 2019).

The nature of this distal pathological seeding was hypothesized by Dr. Heiko Braak. His theory was based on observations that the pathogenesis of NDs, in the context of PD, was mediated by α-Synuclein inclusion bodies that develop first in the gut before translocate to the brain *via* the vagal and motor nerves by an unknown mechanism of gut-brain communication (Braak et al., 2003) (see next section). This hypothesis was then applied to β-amyloid pathogenesis by (Sun et al., 2020). In this study, 2-month-old ICR mice were injected with HiLyte Fluor 555-labelled Aβ42 (the APP isoform most prone to oligomerization) into the stomach and colon and exhibited defects in responsive/exploratory behavioral, short-term and long-term memory cognitive deficits 1-year post-injection. Despite evidence that the labelled Aβ42 monomers did not diffuse through the tissue, Aβ42 remained localized to cholinergic neurons at the injection sites 3 hrs and 3 days post-injection. Further, these mice displayed clear depositions of Aβ plaques throughout the brain, and vagus nerve (DMV), with plaques visible in the hippocampus, cortex, amygdala, and blood vessel walls. Thus, intra-GI administration of Aβ had a direct effect on the neuronal system, with β-amyloid deposits from the gut translocating to the CNS from the ENS *via* the vagal nerve by an unknown mechanism of gut-brain communication. Although this work hypothesized that a retrograde transport route had caused the presence of Αβ aggregates in the brain of the mice, there remains no direct evidence demonstrating that enteric Αβ seeds can retrogradely invade the CNS to induce AD symptoms. However, it is posited that the enteric Aβ seeds invade the brain and cause dementia by retrograde axonal transport through the vagal nerves and haematogenous routes, as seen in prion pathology (Watts et al., 2014). The enteric Αβ seeds did not seem to affect GI major functions when the animals were analysed at 1-year post-injection and only alteration of spontaneous contractions and neuronal couplings of the jejunum, which did not result in constipation, were observed. A difference in contraction frequency of the GI was detected also in another APP transgenic mouse (Semar et al., 2013). Other interesting metabolic defects such as weight gains were observed in various transgenic models. Weight changes (Wagner et al., 2019) have been associated with dementia in humans and are linked to disease progression (Ikeda et al., 2002). In a similar study (Galloway et al., 2007), identified neuronal tissue death alongside translocation of β-amyloid from the gut of mice.

### Systemic oxidative stress as a cause of Aβ protein aggregates in the CNS and pathogenesis

While these murine studies demonstrated that Aβ42 aggregates can form in the GI tract and then enter the brain leading to cerebral amyloidosis and AD-like dementia, other studies showed the existence of a correlation between the amount Aβ42 protein and the extent of oxidative stress and inflammation in the brain of mice and humans (Tamagno et al., 2021). The brain’s vulnerability to oxidative stress is considered a crucial detrimental factor in AD. Aβ induces oxidative stress, and on the other hand oxidative stress can increase Aβ deposition. Notably, while the increase of soluble Aβ42 is correlated to elevated oxidative stress, inflammation, and tissue atrophy of the brain, neither the rate of dementia nor the extent of neurological damage is correlated with the Aβ amyloid. Studies on transgenic mice carrying AD-linked mutations in the gene for APP demonstrated the existence of soluble Aβ oligomers long before the deposition of β-amyloid, further supporting the hypothesis that in particular conditions, an over-production of soluble Aβ aggregates occurs in the human brain even in the absence of plaques. One of the main mechanisms that appears to break this balance is oxidative stress and neuroinflammation (Chang et al., 2014). There is growing consensus that oxidative stress represents a common mechanism that mediates the accumulation and toxicity of Aβ (Saeedi and Rashidy-Pour, 2021). Therefore, oxidative stress can be considered one of the factors responsible for the accumulation of Aβ.

Preliminary evidence from transgenic APP mice and AD human patients indicates that build-up of Aβ in enteric neurons causes inflammation of the gut before any sign of disease is detected in the brain. Enteric inflammation promotes “leaky” guts that release inflammatory mediators/bacteria-derived products into the blood circulation, leading to systemic/neuro-inflammation by weakening the BBB (Chalazonitis and Rao, 2018; Kowalski and Mulak, 2019). For a review of the role of the BBB in neurodegeneration, we refer the readers to (Hussain et al., 2021). Other studies support the theory that oxidative stress and inflammation in the gut may instead trigger β-amyloid aggregation in the brain by means of chronic, low-grade systemic inflammation (Braak et al., 2003). This was proposed from observations that a long period of gradual accumulation of oxidative damage precedes and results in the appearance of clinical and pathological AD symptoms, including Aβ deposition, NFT formation, metabolic dysfunction, and cognitive decline. This suggests that AD begins many years before its symptoms appear, and that antioxidant treatment can be an important therapeutic target to treat the disease (Saeedi and Rashidy-Pour, 2021). Like many neurological disorders, AD is associated with a variety of GI symptoms, raising the possibility that the ENS could also be affected. A clinical report showed that Aβ plaques were found in the submucosa of two AD patients (Joachim et al., 1989). However, with a paucity of evidence and some discordance in the findings, there remains a clear need for further studies of enteric neuronal pathology in AD.

### The microbiota as a trigger of amyloid aggregates and neuroinflammation in the pathogenesis of AD

The stimuli that trigger β-amyloid inclusion formation in the gut remain to be elucidated. The gut microbiota is a significant source of amyloids, changes in the microbiota may potentiate disease in the gut. Disruption of the microbiome occurs as a result of pathogenic infection, antibiotic treatment, aging, and local inflammation (Hakansson and Molin, 2011; Rinninella et al., 2019). Certain bacterial species in the gut, such as *E. coli,* can even produce bacterial amyloids that help the bacteria to form biofilm and survive mechanical and immune stressors (Kowalski and Mulak, 2019). Although bacterial amyloids differ from CNS amyloids in their primary structure, they share similarities in their tertiary structure (Friedland, 2015; Zhao et al., 2015). Shifts in commensal populations that favour species releasing amyloids would increase the concentration of bacterial-derived amyloid proteins in the gut triggering a prion-like cascade of β-amyloid oligomerization/aggregation in the gut (Hetz and Saxena, 2017) or promote cleavage of APP into its Aβ-amyloid peptides (Sarantseva et al., 2009; Bekris et al., 2010; Simon, 2018; Li et al., 2019). A pioneering study of PD carried out by (Chen et al., 2016) demonstrated that rats exposed to amyloid-producing *Escherichia coli* displayed increased neuronal α--Synuclein deposition in both the gut and brain and enhanced microgliosis and astrogliosis compared to rats exposed to bacteria without the ability to produce amyloids. Moreover, in the brain of animals exposed to amyloid-producing bacteria they measured an increased expression of inflammatory cytokines and reactive oxidative and nitrosative stressors which lead to neuronal and glial cell death (Chen et al., 2016; Li et al., 2016). Thus, bacterial amyloids can act as molecular mimics of prion proteins, eliciting cross-seeding, in which one amyloidogenic protein (bacteria amyloid protein) promotes another (e.g., host proteins) to also adopt a pathogenic β-sheet structure (Lundmark et al., 2005; Zhou et al., 2012).

Therefore, a combination of gut inflammation and dysbiosis is directly associated with gut barrier dysfunction and increased intestinal permeability (“leaky gut”) may contribute to the process of neurodegeneration (Marizzoni et al., 2017; Sochocka et al., 2019). Lipopolysaccharide (LPS) from commensals or pathogenic bacteria leaked from the gut can reach and accumulate in the brain and activate microglia, brain resident immune cells, and elicit inflammatory responses in the brain. LPS has been detected in the hippocampus and neocortex brain lysates from AD patients and has been found to colocalize with Aβ40/42 β-amyloid plaques (Zhao et al., 2017). Interestingly, the plasma concentration of LPS in AD patients is significantly higher than in healthy people (Zhang et al., 2009), and repeated systemic exposure to LPS in mice induced microglial priming and prolonged cytokine production.

Perturbation in microglia activation has been identified as a feature of NDs progression. For full reviews on the topic, we refer the readers to (Hickman et al., 2018) and (Muzio et al., 2021).

### Tao-derived aggregates in the gut in the pathology of AD

Tauopathies, are a group of NDs that include a form of AD, which are characterized by abnormal hyperphosphorylation of microtubule-associated protein tau that leads to the formation of NFTs. Similar to β-amyloid pathogenesis of NDs in the brain, tau monomers oligomerize and form aggregates known as NFTs and neuritic plaques, which form inclusion bodies that cause inflammation due to intra/extra-cellular damage in and around neurons (Anwal, 2021; Bălaşa et al., 2020; Takatori et al., 2019). NFTs have been found to accumulate in the gut and to cause gastrointestinal dysfunction such as alteration of gut motility before any clinical symptom is found in the brain (Kametani and Hasegawa, 2018). Moreover, both β-amyloid and also tau tangles/plaques promote local intestinal inflammation and weakening of the gut epithelial barrier (Kowalski and Mulak, 2019). The weakening of the gut epithelial barrier can cause metaflammation (low-grade chronic systemic inflammation) that has been linked to NDs (Komleva et al., 2021; Rydbom et al., 2021). For a review on metaflammation we refer the readers to (Itoh et al., 2022).

Although the mechanisms of DMGB axis pathogenesis in the context of ADs are poorly defined, concurrent evidence shows that the diseases may manifest/originate in the gut before appearing in the brain.

## The gut-brain axis in parkinson disease

### Physiopathology of parkinson disease

PD has been identified as one of the most rapidly growing NDs in the world (Dorsey et al., 2018) and is more prevalent in males than females (Moisan et al., 2016; Podcasy and Epperson, 2016; Feigin et al., 2019). This disease is primarily defined by progressive loss of motor-function (initiation and coordination) of the brain, along with bradykinesia. These behavioral disturbances are due to atrophy of the substantia nigra (Faustini et al., 2017; Dorsey et al., 2018), an essential medullary brain region that produces and projects dopamine to the limbic system, and higher cortical areas (Mayfeild, 2010). These projections are known as nigrostriatal pathways, activating regions of the brain that control motor-behavior (Zeighami et al., 2015; Faustini et al., 2017; Sonne, et al., 2021). Despite a similar pattern of atrophy in PD brains compared to healthy aging brains, the pathologies of the disease are what exacerbate and accelerate this phenotype (Zeighami et al., 2015). Hallmark pathologies of PD in the brain include intracellular misfolded protein aggregates of α-Synuclein oligomers (synucleinopathies) that form plaques called Lewy Body inclusions in the substantia nigra (Huang et al., 2015; Wang et al., 2016; Shahmoradian et al., 2019). These intracellular inclusions cause aberrant neurotransmission because, α-Synuclein is a SNAP-associated protein in neurons of the CNS, PNS and ENS that tethers and primes pre-synaptic vesicles for exocytosis (Killinger et al., 2019). Considering the similar effects of tau and β-amyloid to α-Synuclein proteins in neurotransmitter trafficking along the axon and release, it is not surprising that PD and AD are comorbid. Especially, since unresolved misfolded proteins with prion-like effects are characteristic of both diseases (Xie et al., 2014).

### Evidence of the seeding of parkinson disease in the gut prior to the brain

**FIGURE 2:**
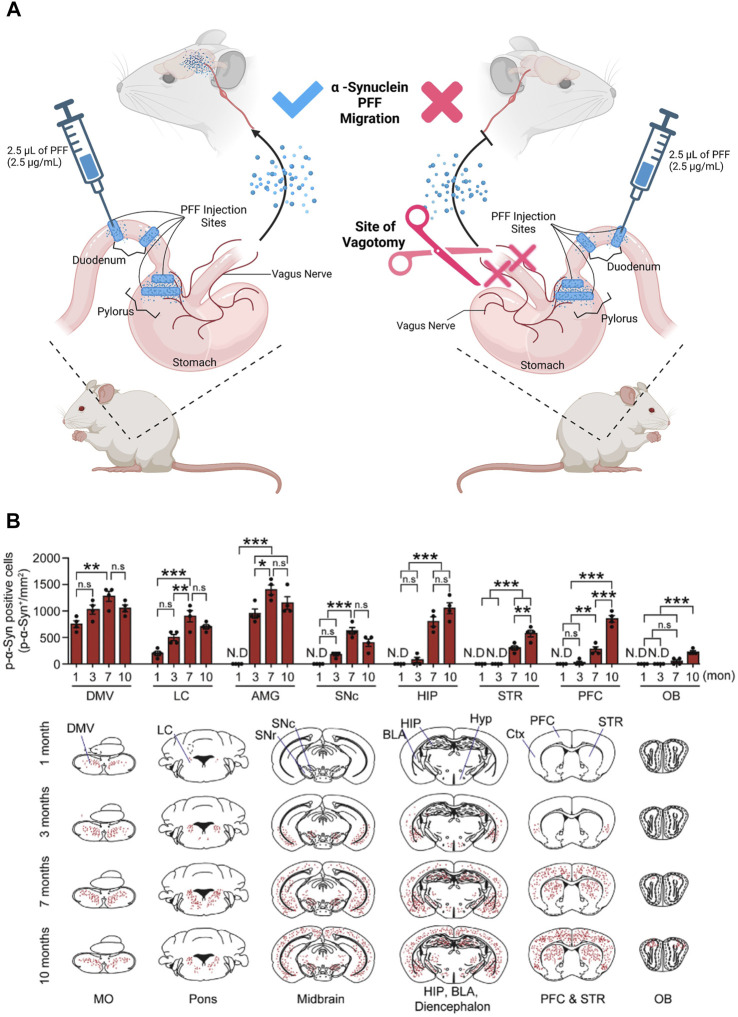
α-Synuclein progressively translocates from the gut into the brain: experimental diagram and result. **(A)** PFFs Pathologic α -Synuclein (pSer129- α -syn), as Pre-Formed Fibrils (PFFs), were injected into the duodenum and pylorus of a mouse. Translocation of Pre-Formed Fibrils (PFFs) of Pathologic α-Synuclein from the uppermost division of the small intestine was facilitated by the nodose ganglion (the vagal nerve afferents of the mouse). To confirm the credibility of this migratory route, the anterior trunk of the vagal afferents were severed (site of vagotomy). PFFs were not identified in the brains of the mice that received an anterior trunk vagotomy. **(B)** The graphs represent the progressive translocation of PFFs from the gut into the brain after 1–10 months from the injection. At month one post injection, the α-Synuclein PFFs translocates into the brain, first appearing at the Medulla Oblongata (MO). The trans-neuronal propagation and migration of the PFFs into the mouse brain proceeded from the posterior MO to the mouse brain’s anterior Olfactory Bulb (OB) (MO to OB). Within 1 month post injection, PFFs were detected in the Dorsal Motor Nucleus of the Vagus nerve (DMV) of the MO, and Locus Coeruleus (LC) of the Pons. At 3 months post injection, the abundance of PFFs in the Amygdala (AMG) spiked, while trace amounts of PFFs in the substantia nigra para compacta (SNc), Hippocampus (HIP), and Pre-Frontal Cortex (PFC) were detected. At 7 months post-injection, PFFs were detected in the Striatum (STR) and olfactory bulb (OB). Finally, at 10 months PFF has infiltrated and diffused through the entire brain. Data extracted and modified from (Kim et al., 2019). Figure constructed on BioRender.com.

Recent works demonstrated that, α-Synuclein can be detected in the ENS years before clinical onset of PD, similar to tau and β-amyloid (Hawkes et al., 2010; Shannon et al., 2012; Devos et al., 2013; Driver-Dunkley et al., 2014; Hilton et al., 2014; Iranzo et al., 2014; Stokholm et al., 2016). This α-Synuclein accumulation causes constipation and inflammation of the gut that often leads to Inflammatory Bowel Disease (IBD) (Barbut et al., 2019; Brudek, 2019; Chen et al., 2019). As mentioned in the previous section, studies in PD rodent models have shown that α-Synuclein seeded in the enteric neurons of the colon, duodenum, and stomach (Shannon et al., 2012; Sánchez-Ferro et al., 2015; Kim et al., 2019; Challis et al., 2020) migrate *via* the vagal nerve to the brain (Braak et al., 2003; Holmqvist et al., 2014; Uemura et al., 2018). This was demonstrated in experiments where the α-Synuclein-rich lysate, prepared from severely affected PD patient substantia nigra brain tissue, was injected into the intestines of wild type rats leading to the detectable accumulation of α-Synuclein over time in both the DMV and the brain (Holmqvist et al., 2014). This study also showed that α-Synuclein was selectively up-taken by the DMV compared to BSA. Notably, α-Synuclein accumulated at the Dorsal Motor nucleus of the vagus nerve (DMV), a collection of cholinergic neurons. Furthermore, DMV migration of α-Synuclein was prevented by a vagotomy of the base-afferent vagal nerve, as demonstrated by (Kim et al., 2019) (Figure 2). Therefore, cholinergic neurons must facilitate long-distance translocation of α-Synuclein (monomer, oligomer, and fibril) *via* a microtubule-associated transport mechanism (Holmqvist et al., 2014). By this notion, the question that surfaces is whether the α-Synuclein inclusions in the DMV are the reason behind the GI issues observed in PD patients, or if the symptoms are the reason for the aggregation of α-Synuclein, exacerbating symptoms and pathology in the DMV.

Several studies have confirmed the migration of α-Synuclein from the ENS (Challis et al., 2020), by tracking fluorescently tagged-α-Synuclein, inoculated into the intestines of mice. The tagged-α-Synuclein translocated from the gut to the brain, and created human Parkinson-like symptoms, that affected sensorimotor behaviors including diminished ability of mice to climb down a pole or hang from a metal wire for an extended period of time. In addition, abnormalities in GI function as well as inflammation were reported in mice that received an intestinal injection of tagged-α-Synuclein. This recapitulated symptoms experienced by pre-clinical human PD patients (i.e. constipation and bowel inflammation) (Dinan and Cryan, 2017; Challis et al., 2020). Therefore, α-Synuclein pathogenesis can be seeded in the gut and ultimately, *via* the gut-brain axis, move to the brain. However, we still need to understand what triggers the accumulation of α-Synuclein in the intestine and leads to pathogenesis.

### Implication of diet and microbes in the pathology of PD

Since IBD and therefore intestinal inflammation occurs in PD patients before the brain pathologies begin, diet and commensals are suspected to play a role in the formation of α-Synuclein Lewy bodies (Rinninella et al., 2019; Mertsalmi et al., 2020). Mitochondrial abnormalities are observed in the gut of IBD (Jackson and Theiss, 2020), and PD (Brudek, 2019) patients. Protein α-Synuclein aggregates have been found to cause mitochondrial damage by interacting with complex-I of the electron transport chain, and slowing down mitochondrial metabolism (Kamp et al., 2010; Faustini et al., 2017). This type of mitochondrial stress increases the redox state of intestinal epithelial cells and creates inflammation (Friedman and Nunnari, 2014; Console et al., 2020). Therefore, α-Synuclein in PD might act just like β-amyloids and tau in AD, as a facilitator and amplifier of PD by seeding pathology in the gut, ultimately affecting the brain (Braak et al., 2003; Kitani-Morii et al., 2021). As for AD, the pathology in the gut could be influenced by oxidative stress and tissue inflammation of the gut that enhance protein aggregate formation and/or gut permeability that is translated to the brain, causing neuronal death and impairment of memory and locomotion. Thus, protein α-Synuclein aggregates in the gut can transfer to the brain or can generate local inflammation that distally impacts the BBB and neuronal activity as reported of Aβ amyloids.

Additionally, several genome-wide association studies revealed that mutations in leucine-rich repeat kinase 2 (LRRK2) that are one of the greatest genetic contributors to PD are also linked to increased incidence of Crohn’s Disease (CD), a form of IBD (Hui et al., 2018). Although to date, the pathology of CD does not include neuropathology, CD is characterized by an increase in leakage of the epithelium, inflammation, and dysbiosis in the GI, all features that seem to contribute to neuroinflammation and neuropathology in PD. Therefore, further studies looking at the role of LRKK2 in CD could help to define the mechanisms of the disease of PD and the role of gut-brain communication in the PD pathogenesis.

Overall, NDs such as AD and PD do not arise exclusively from defects in the brain, but signals coming from peripheral organ environments and inter-organ interaction can wire messages to the brain and effect the brain. Therefore, more studies are required to dissect the mechanisms of action of the DMGB in health and NDs. Fortunately, the use of multidisciplinary approaches and more amenable model organisms will fast-track our knowledge in this field.

## Model systems to study the DMGB axis in neurodegenerative diseases

Recent efforts to unravel the diet-microbiota-gut-brain networks have relied on genome-wide association studies and metagenomic data in small cohorts of human samples to identify disease-associated factors. It is now clear that to dissect the mechanism underlying such multifactorial diseases, the use of multidisciplinary approaches with heavily controlled, reductive model systems is an enormous asset. Various model systems have been developed to study NDs primarily in the brain (see table 1), and currently being developed for the gut to study the gut-brain axis (discussed below).

**TABLE 1:** Models of *Mus musculus* (Mouse) and *Drosophila melanogaster* (Fruit fly) for studying the human neurodegenerative disease. The reported models represent Tauopathies, β-amyloidopathies, and Synucleinopathies in the brain. Models include Knock-out (KO), Knock-in (KI), Knock-Down (KD), and Over-Expression (OE) of hallmark genes for each of the three categories of neurodegenerative disease listed above. There is a column describing similarities in pathology between species and another column for differences. With each example made, there are references to the literature for more information about the model organism’s pathologies and similar or contrasting human pathology for comparison.

Target gene- manipulated in the brain	Neurodegenerative disease classification	Comparing with human disease	Contrasting with human disease
***dTau* (KD) In *Drosophila***	**Tauopathy**	**Conservation of structure:** *Drosophila*-Tau (dTau) is 46% identical, 66% similar to human-Tau (hTau). Heidary and Fortini (2001)	dTau in the fly is the only member of Tau/Map2/Map4 family identified in humans
		**Conservation of function:** hTau expression in dTau (KD) model rescues neurodegenerative phenotypes	Heidary and Fortini (2001), Dehmelt and Halpain (2005)
		Retinal and central nervous system degeneration (Vacuole formation) Ho et al. (2012); Bolkan and Kretzschmar (2014)	Conditional fibrillary tangle formation upon co-expression of dTau with human Tau kinases Wittmann et al. (2001)
		Rounded axons less tightly packed together Raff et al. (2002); Bolkan and Kretzschmar (2014)	
***hTau* (Human Mutant Tau R406W) (OE) In *Drosophila***	**Tauopathy**	**Conservation of function:** Age-dependant onset of progressive neurodegeneration observed in the cortex and neuropil resembling the progressive stages of AD Wittmann et al. (2001); Braak et al. (2003)	No appearance of NFTs Lee et al. (2001); Wittmann et al. (2001); Fu et al. (2017)
		Degeneration of cholinergic neurons Wittmann et al. (2001); Mesulam et al. (2004); Cranston et al. (2020)	Vacuolization (like holes in Swiss Cheese) is not a prominent phenotype of human NDs, but is commonly observed in *Drosophila* neurodegeneration Kretzschmar et al. (1997)
		Accumulation of hyperphosphorylated tau Grundke-Iqbal et al. (1986); iqbal et al. (2010); Spillantini and Goedert (2013); Wittmann et al. (2001)	
		Reduced longevity Wittmann et al. (2001); Strand et al. (2018)	
***mTau* (KO) In *Mus***	**Tauopathy**	**Conservation of structure:** Mouse-tau **(**mTau) is 92% homologous at the c-terminus, and 57% homologous overall with hTau Adams et al. (2009)	Tau isoform expression in adult mice differs from human Brion et al. (1993); Lei et al. (2012); Spillantini and Goedert (2019); Hernández et al. (2020)
		**Conservation of function:** Decline in locomotor function and range of movement with age Lei et al. (2012), a characteristic of certain human tauopathies such as frontal lobe dementias with motor neuron disease Neary et al. (1990); Lei et al. (2012); Audouard et al. (2015)	No overt phenotype until aged, redundancy of other MAPs that can substitute for tau. Harada et al. (1994); Lei et al. (2012)
		Reduced velocity, shorter strides, average displacement in an open environment Nadkarni et al. (2009); Lei et al. (2012)	
		Fewer substantia nigra dopaminergic neurons and less striatal dopamine abundance Lei et al. (2012); Roostaei et al. (2017); Nam et al. (2018)	
		Administration of L-DOPA helps alleviate motor symptoms Lei et al. (2012); Turcano et al. (2020)	
		Brain atrophy: Reduced brain mass, and neocortical shrinkage Thompson et al. (2003); Lei et al. (2012); Schäfer et al. (2021) and ventricular enlargement Lei et al. (2012); Dugger et al. (2017)	
		Cognitive loss: Accounted for by impaired ability to recognize familiar spaces and willingness to explore new areas (Apathy) Neary et al. (1990); Lei et al. (2012)	
		Reduced abundance of BDNF in the hippocampus (Lei et al. (2012); Jiao et al. (2016)	
***htau***_***P301L***_ **(Mutant human-tau) (OE) In *Mus***	**Tauopathy**	**Conservation of function:** The Accumulation of hyperphosphorylated Tau and NFTs in cortical neurons with aging Lee et al. (2001); Santacruz et al. (2005); Fu et al. (2017)	
		Loss of hippocampal neurons Santacruz et al. (2005); Kovacs et al. (2018)	
		Decline of brain matter as measured by reduced weight Thompson et al. (2003); Santacruz et al. (2005); Schäfer et al. (2021)	
		Age dependant impairment of spatial memory. Santacruz et al. (2005); Lithfous et al. (2013); Fu et al. (2017)	
		Deficits in spatial navigation Santacruz et al. (2005); Lithfous et al. (2013); Allison et al. (2016)	
***hAß42* (KI) In *Drosophila***	**β-amyloidopathy**	**Conservation of function:** Expression of human-Aβ-42 (hAß42) and extracellular secretion results in mimicked AD effects seen in the human Iijima et al. (2004); Murphy and LeVine (2010); Yoo et al. (2020) and *Drosophila* have conservation of γ-secretase activity Fossgreen et al. (1998); Takasugi et al. (2003)	No β-amyloid fibril formation Wittmann et al. (2001); Iijima et al. (2004)
		Amyloid deposits Iwatsubo et al. (1994); Gravina et al. (1995); Iijima et al. (2004)	*Drosophila* APP homolog (d APP) does not have Aβ domain to be cleaved (endoproteolysis of) for flies. Rosen et al. (1989); Martin-Morris and White (1990); Iijima et al. (2004)
		Age-dependent learning defects: Decline in olfactory learning, and memory (Working/short term) Iijima et al. (2004); Villemagne et al. (2008)	No homolog to beta-secretase Fossgreen et al. (1998); Iijima et al. (2004))
		Locomotor decline Iijima et al. (2004); Pottorf et al. (2022)	
		Brain atrophy: Dying/necrotic neurons in the Kenyon cell layer (part of the mushroom body, analogous to human hippocampus) Iijima et al. (2004); Pini et al. (2016))	
		Mitochondrial defects Iijima et al. (2004); Wang et al. (2020)	
***hBACE,* and *hAPP* (OE) In *Drosophila***	**β-amyloidopathy**	**Conservation of function:** Age-dependant plaque formation when human-BACE and human-APP (hBACE, and hAPP) are expressed Yamaguchi et al. (1992)	Plaque formation more severe in male than in female flies. Opposite observed in humans. Yamaguchi et al. (1992); Podcasy and Epperson (2016)
		Degeneration of the retinal photoreceptors Yamaguchi et al. (1992); Ratnayaka et al. (2015)	
		Age dependent progression of neurodegeneration and EM ultrastructure with star-like formation resembles human AD plaques Yamaguchi et al. (1992); Walker (2020)	
***hAPP*** (***V717F***) **(OE) In *Mus***	**β-amyloidopathy**	**Conservation of structure:** Overall, the mouse - APP (mAPP) is approximately 52.7% homologous to the human- APP (hAPP) von der Kammer et al. (1994)	The absence of NFTs Games et al. (1995); Wittmann et al. (2001)
		**Conservation of function:** Mice have a native gamma and beta secretase Games et al. (1995)	
		Deposits of hAβ in the hippocampus, and cerebral cortex Games et al. (1995); Pini et al. (2016); Svenningsson et al. (2019)	
		Synaptic and dendritic density reduced in hippocampal dentate gyrus Games et al. (1995); Pini et al. (2016); Svenningsson et al. (2019)	
		Extracellular β-amyloid legions surrounded by reactive astrocytes similar to gliosis observed in AD Canning et al. (1993); Games et al. (1995)	
***mAPP* (KO) In *Mus***	**β-amyloidopathy**	**Conservation of function:** No evidence of cortical abnormalities such as swelling and atrophy until aged, when mouse-APP (mAPP) is knocked down Zheng et al. (1995); Pini et al. (2016)	No immunoreactive neurons or gliosis around the hippocampal and cerebellar neurons Canning et al. (1993); Zheng et al. (1995)
		Aβ deposits localized to the hippocampus and cerebral cortex Zheng et al. (1995); Pini et al. (2016); Svenningsson et al. (2019)	
		Decreased locomotor activity Zheng et al. (1995); Pottorf et al. (2022)	
***hLRRK2* (OE) In *Drosophila***	**Synucleinopathies**	**Conservation of Function:** Bradykinesia, akinesia, hypokinesia, and tremors when human-LRRK is expressed (hLRRK2) Kaasinen et al. (2014); Cording et al. (2017)	There is no a-Synuclein homolog in the fly
		Slow movement and tremors in the proboscis of the fly Berardelli et al. (2001); Cording et al. (2017)	
		Rescue of symptoms with L-DOPA and kinase inhibitors Growdon et al. (1998); Cording et al. (2017)	
**α -Synuclein (*hSNCA*) (OE) In *Drosophila***	**Synucleinopathies**	**Conservation of function:** Adult onset of dopaminergic neuronal cell death, when human α -Synuclein is expressed (hSNCA), no matter expression of WT or mutant hSNCA Feany and Bender (2000); Poewe et al. (2017)	No loss of volume in the brain or central neuropil, and no vacuolization as observed with other *Drosophila* models of NDs
		Cytoplasmic inclusions resembling Lewy Body plaques and tangles for both WT and mutant α -Synuclein OE	
		Deficits in negative geotactic response with aging (locomotor deficit) Feany and Bender (2000); Berardelli et al. (2001); Poewe et al. (2017)	
**α -Synuclein (*mSNCA*) (KO) In *Mus***	**Synucleinopathies**	**Conservation of structure:** 95% of the α -Synuclein sequence is conserved between the mouse and human, however, confirmation of secondary structure is different, and determines aggregation likelihood Kang et al. (2011)	No motor symptoms with aging, however suggested anxiety-related phenotype
		**Conservation of function:** Early signs of neuroinflammation when mouse α -Synuclein is knocked out (mSNCA): Microglia were hyper reactive with vacuole-like structures intracellularly Cabin et al. (2002); Armstrong (2017); Guillot-Sestier and Town (2018)	No α -Synuclein Lewy Body pathology Dijkstra et al. (2014)
		Mitochondrial abnormalities in the electron transport chain complex I/III and reduced cardiolipin phospholipid in mitochondrial membrane Cabin et al. (2002); Ordonez et al. (2018)	No definite reduction in striatal dopamine levels (variable within KO mice)
		Abnormalities in synaptic neurotransmission regarding replenishment and smaller neurotransmitter reserves of vesicle pool required for repeated simulation Cabin et al. (2002); Benskey et al. (2016)	No gross neuronal cell death Dijkstra et al. (2014); Poewe et al. (2017)
***mLRRK2* (OE) In *Mus***	**Synucleinopathies**	**Conservation of function:** Abnormalities in the nigrostriatal system when human-LRRK2 (hLRRK2) is expressed (No matter a wild type or mutant allele), including the decreased dopamine release and locomotion Gehrke et al. (2010); Poewe et al. (2017); Coon and Singer (2020)	Minimal evidence of neurodegeneration No α -Synuclein Lewy Body pathology Gehrke et al. (2010); Dijkstra et al. (2014); Coon and Singer (2020)
		Rescue of symptoms with L-DOPA Growdon et al. (1998); Gehrke et al. (2010)	Pleomorphic pathology in LRRK2 parkinsonism (Hasegawa et al., 2009; Gehrke et al. (2010)

### The murine model system to study NDs

The mouse mammalian model system in particular presents 90% synteny to the human genome (Monaco et al., 2015) and has been used in over 95% of studies of human disease (Vandamme, 2014). Mouse models of NDs have facilitated the study of neurodevelopmental and neurological diseases, and advanced our understanding of the molecular pathogenesis of NDs in humans (Dawson et al., 2018; Zhao and Bhattacharyya, 2018). In particular, the models that produce similar ND pathologies within the most conserved region of the mouse brain, the neocortex, have been extensively used (table 1). For example, transgenic or knockout (KO) mouse models for tau, β-amyloid, and α-Synuclein (table 1) have been used to investigate the physiological roles of the familiar forms of PD genes and to model dopaminergic degeneration caused by the dysfunction of these genes. Although these models exhibit various pathological and behavioral phenotypes, some aspects of PD pathogenesis such as the degeneration of distinct dopaminergic neurons or NFTs have not been reproduced (Harada et al., 1994; Cabin et al., 2002; Hasegawa et al., 2009; Chen et al., 2022).

**FIGURE 3:**
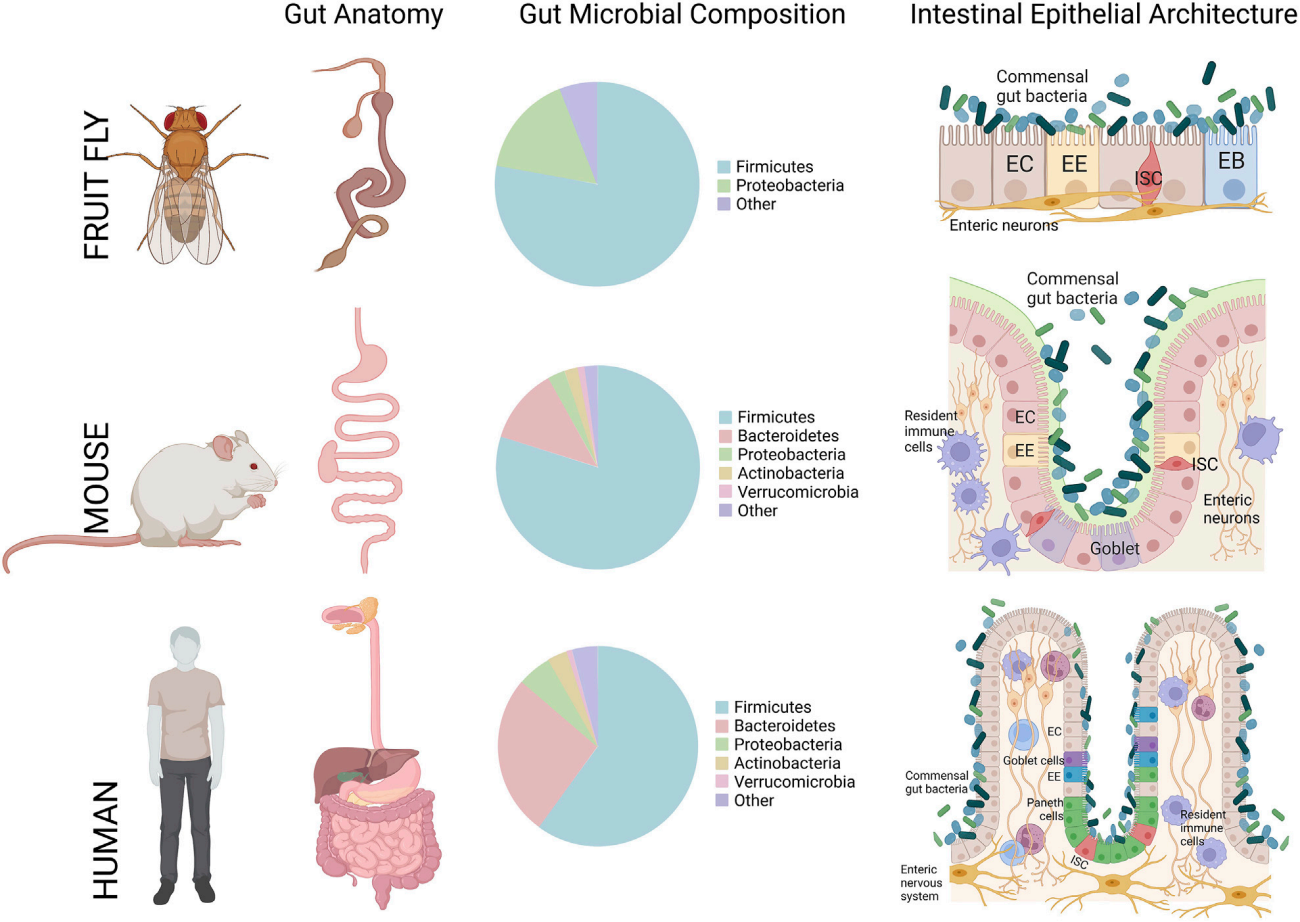
A cross-comparison of the gastrointestinal tract gross anatomy, gut microbiota composition, and general intestinal epithelial architecture between *Drosophila melanogaster* (Fruit fly), *Mus musculus* (Mouse), and *Homo sapiens* (Humans). In the small intestine, for mouse and human, and midgut, for the fruit fly, all three species share *Firmicutes* and *Bacteroidetes* microbial population as the dominant resident phyla. Figure constructed on BioRender.com, pie charts of common phyla that compose the gut-microbiota are referenced from (Trinder, et al., 2017).

In context of the intestinal epithelium, mice have also been extensively used in biomedical disease studies of intestinal dysplasia and inflammation such as IBD, or infection models to better understand the pathogenesis of gut-borne pathogens (Gkouskou et al., 2014; Vandamme, 2014; Stanford, et al., 2020). The fundamental gross/cellular anatomy of the mouse gut is also very similar and has been considered one of the best comparable animal models to the human gut (Figure 3) (Trinder et al., 2017; Stanford et al., 2020).

With regards to studies defining the effect of microbiota on animal physiology, the mouse has one of the most similar gut-commensal populations to the human when comparing the presence and abundance of major phylogenetic groups, such as *Firmicutes*, and *Bacteroidetes* (Figure 3) (Xiao et al., 2015; Trinder et al., 2017). However, studying the highly-intertwined DMGB axis is more challenging in a complex mammalian system where the cost and infrastructural hurdles of constructing germ-free mouse models is high. Moreover, it is very difficult to control multiple factors while performing experiments where environmental changes may greatly influence the results (Trinder et al., 2017). These challenges together with the high cost and time required to generate these models add further risk to the use of such models given the overarching difficulty in knowing if the phenotypes observed accurately recapitulate disease states in humans (Vandamme, 2014). These issues have prompted researchers to incorporate alternative model organisms for these studies.

#### An emerging model system to study DMGB axis: *D. melanogaster*

**FIGURE 4:**
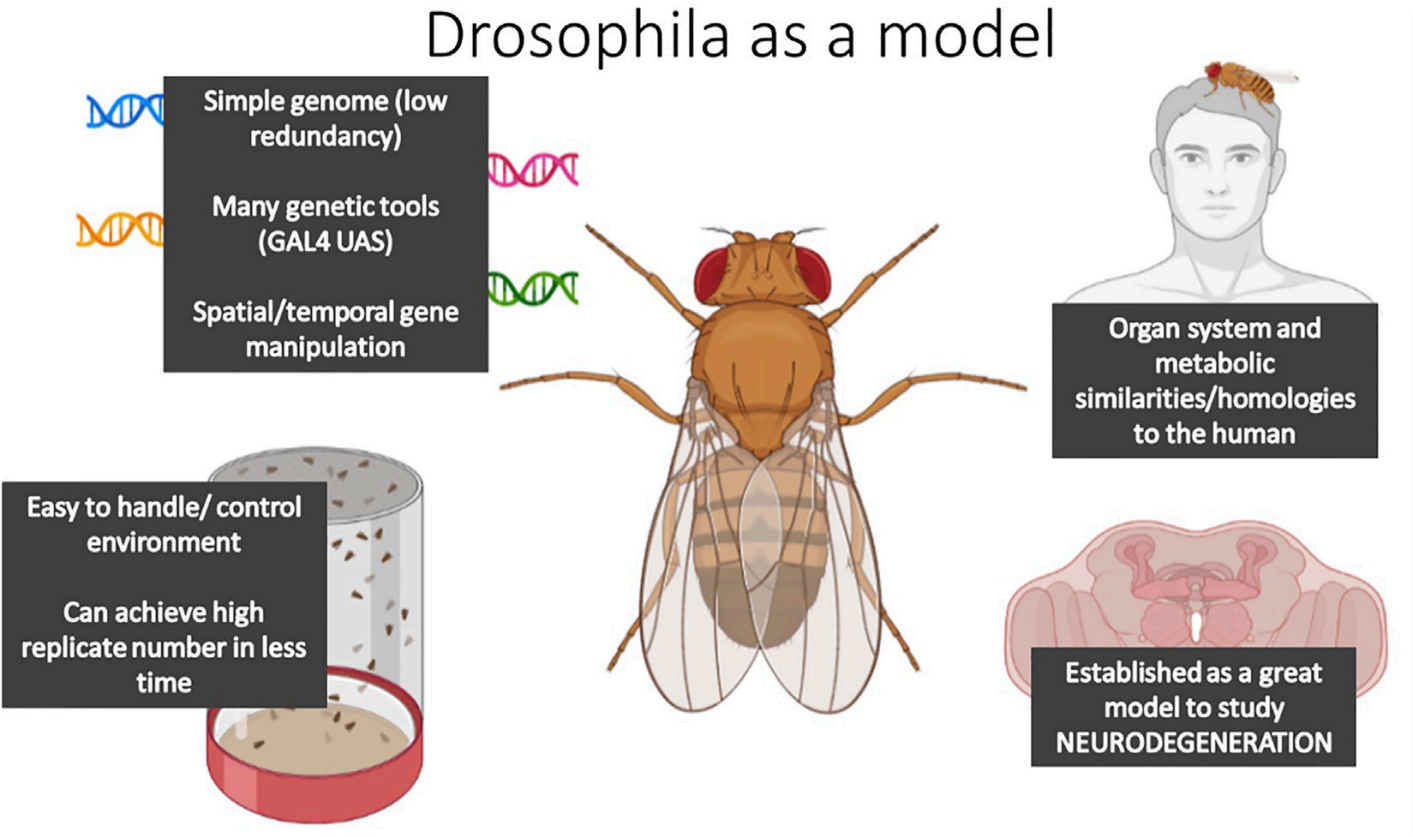
The diagram summarizes the main features that make *Drosophila melanogaster* a suitable model organism to study DMGB axis.

In the last 10 years, the *Drosophila* model system has been affirmed as a valid model organism to unravel the mechanisms underlying NDs. *Drosophila* share similar gross organ function and inter-organ communication networks with mammalian systems (Figure 3; Figure 4) (Prüßing et al., 2013; Cassim et al., 2018; Jahromi et al., 2020)*,* and carry many conserved genetic, cellular, and metabolic factors with mammals (Figure 4) (Cassim et al., 2018). For example, the nervous system of the fly has functionally analogous brain regions to the mammalian brain, such as the mushroom body for learning and memory, analogous to the mammalian hippocampus, the blood-brain barrier and the central complex for movement; comparable to the basal-ganglia and precentral gyrus in humans (Barnstedt et al., 2016; Gomez-Marin et al., 2016). Moreover, the nervous system of both flies and mammals shares similar mechanisms of neurotransmission, i.e. dopamine in dopaminergic neurons of the central complex in *Drosophila* facilitate locomotion, as the basal ganglia does in mammals (Martin and Krantz, 2014). Moreover, studying NDs in *Drosophila* allows studies where the environment can be easily controlled, large populations can be easily obtained and analyzed at lower costs, and where complex networks can be quickly dissected due to the availability of elegant genetic tools (Figure 4). Transgenic AD and PD models, that present similar behavioral/cellular pathologies to human NDs, have been successfully constructed expressing canonical human-linked alleles of NDs such as α-Synuclein and β-secretase. Furthermore, the characterization of these models has yielded great advances into the basis of neurodegenerative disease etiologies (Jeibmann and Paulus, 2009; Lu and Vogel, 2009; Perrimon et al., 2016; Tan and Azzam, 2017; Cassim et al., 2018; Xiong and Yu, 2018), therefore, making the human disease easier to study in less complex, but analogous, organism.

Core immuno-inflammatory pathways such as Toll, JAK/STAT, and immunodeficiency (Imd) equivalent to TLR-4, JAK/STAT and TNF pathways in mammals (Hoffmann and Reichhart, 2002; Hoffman, 2003; Myllymäki et al., 2014) are also present in *Drosophila*. These innate immune-inflammatory pathways, whether in humans or *Drosophila,* respond to infection and aging/injury by promoting the expression of inflammatory mediators such as cytokines and antimicrobial peptides (AMPs in fly and humans) (Myllymäki et al., 2014). During the progression of NDs in humans, the release of inflammatory mediators by the innate immune cells (e.g. microglia) leads to systemic inflammation that ultimately can contribute to inflammation of the brain and neuronal damage (Lye and Chtarbanova, 2018; Arora and Ligoxygakis, 2020). Typically, AMPs during infection are neuroprotective, but during aging their constitutive expression in the brain elicit neurotoxic effects that might trigger/exacerbate neurodegeneration (Cao et al., 2013; Kounatidis et al., 2017; Lye and Chtarbanova, 2018). Accumulation of these inflammatory molecules during aging is not necessarily caused by local secretion of AMPs in the brain but can come from peripheral organs. For example, a study carried out in the intestinal epithelium of *Drosophila* showed that over-aging PINK1-mutant flies (PD model), exhibit accumulation of damaged mitochondria in intestinal epithelial cells that activate NF-kB and expression of AMPs that lead to gut dysfunction (Fedele et al., 2020). The high AMP expression leads to age-dependent neurodegeneration. Genetic suppression of NF-kB activity in aging *Drosophila* intestines improved the neurodegeneration phenotype.

In another recent study, the authors examined the role of AMPs on fly’s lifespan over aging. They found that while ablation of individual AMP genes does not impact the animal lifespan during aging, on the other hand, the lack of 14 AMP genes impacts lifespan over aging. Hanson and Lemaitre demonstrate that AMPs control the gut microbiota over aging and protect animal survival, thus demonstrating the importance of gut-microbiota interaction during animal aging (Hanson and Lemaitre, 2022).

These studies strengthened the validity of *Drosophila* as a model system to dissect the mechanism of the microbiota-gut-brain axis in NDs. Moreover, the *Drosophila* GI tract has been researched over decades in regard to interorgan communication and inflammation (Capo et al., 2019; Hung et al., 2020; Okamoto and Watanabe, 2022). The blueprint of the intestine is functional and structurally analogous to humans (Figure 3). For a complete review of *Drosophila*’s intestine we refer the reader to (Capo et al., 2019). The *Drosophila* midgut epithelium, functionally analogous to the mammalian small intestine, is composed of four different cell types. Progenitor cells are intestinal stem cells, and undifferentiated intestinal stem cell daughters referred to as enteroblasts can differentiate into enterocytes, or into enteroendocrine cells in response to diverse and incompletely defined differentiation signals (Figure 3). Among these cells, the enteroendocrine cells function to integrate gut environmental signalling such as dietary nutrients and microbiota-derived metabolites, secrete peptides and cytokines and regulate various physiological processes. Several enteroendocrine cell-derived peptides act locally to regulate gut motility (Okamoto and Watanabe, 2022), proliferate intestinal stem cells (Amcheslavsky et al., 2014; Takeda et al., 2018), modulate lipid metabolism in enterocytes (Song et al., 2014; Kamareddine et al., 2018), and release endocrine factors for interorgan communication. Genetic experiments strongly suggest effects of several enteroendocrine-derived peptides on remote target organs or tissues such as the CNS (Kubrak et al., 2022).

Research with *Drosophila* has also contributed to our understanding of the importance of the microbiota homeostasis for an organism’s health (Douglas, 2018). In both *Drosophila* and humans, gut microbiota abundance and diversity are dependent on the host’s diet (Chaston et al., 2014), host’s immune system (Marra et al., 2021), gut morphology (Broderick et al., 2014), health status and hosts genotype (Clark et al., 2015; Fischer et al., 2017; Téfit and Leulier, 2017). Although the gut-commensal profile of *Drosophila* harbors a lower microbial diversity compared to that found in the mammalian gut (Bakula 1969; Chandler et al., 2011; Corby-Harris et al., 2007, with <30 taxa in the fly (Newell and Douglas, 2014), there are still many phylogenetically common species shared with humans (i.e. *Firmicutes*, the most abundant phyla in both species) (Figure 3), which makes this animal an amenable model to study fundamental gut-commensal interactions (Newell and Douglas, 2014; Trinder et al., 2017; Douglas, 2018). Interestingly, these bacterial species display similar health-promoting proprieties in *Drosophila* and mammals (Erkosar et al., 2013). For instance study of microbiota in transgenic fly models for NDs have revealed correlation between shift in microbial population and aging (Kitani-Morii et al., 2021; Neophytou and Pitsouli, 2022; Schretter, 2020; von Frieling et al., 2020) or the onset of NDs similar to that observed in humans (Mertsalmi et al., 2020; Rydbom et al., 2021).

Considering these benefits, *Drosophila* could complement the information gathered from the murine models of NDs and can be considered one of the most versatile model organisms to study the DMGB axis in the context of diseases such as NDs and interorgan communication.

## The diet-microbiota-gut-brain axis drives neurodegeneration in mice and the flies

The diet-microbiota-gut-brain axis is a rapidly expanding area of research suggesting that the interaction between the intestine, gut-commensals, and diets define the gut-brain signaling by means of neuronal, endocrine, immune, and humoral links that overall impact the brain (Carabotti et al., 2015; de Wouters d’Oplinter et al., 2021; Kitani-Morii et al., 2021). In the following section, we will summarize each component of the DMGB axis and will report results from studies that have established how dysfunction of this axis might be an early sign for the onset of NDs.

## The diet-microbiota-gut-brain axis: The diet

Nutritional replenishing of our energy expenditure impacts numerous aspects of our physiology and dictates the level of individual protection or risk factors for developing diseases such as NDs (Cena and Calder, 2020). Clinical and animal studies highlight the importance of dietary lipids throughout life from neural development to aging and neurodegeneration (Calon and Cole, 2007; Belkind-Gerson et al., 2008; Innis, 2008; Bousquet et al., 2009; Barrett et al., 2014; Chianese et al., 2018; Stavrinou et al., 2020).

Dietary fatty acids can directly affect the brain by crossing the BBB and subsequently changing the neural membrane composition (Chianese et al., 2018). A diet rich in mono and polyunsaturated fatty acids (monounsaturated fatty acids and PUFAs), is associated with anti-inflammatory properties (Tosti et al., 2018). Long-chain dietary n-3 PUFAs, eicosapentaenoic acid, and docosahexaenoic acid, play a regulatory role in immunological responses. They suppress genes involved in inflammation and change the cell membrane composition by displacing n-6 PUFA and cholesterol (Scaioli et al., 2017). Consequently, they alter lipid raft aggregation and affect cell signaling (Scaioli et al., 2017). Eicosapentaenoic acid, docosahexaenoic acid, cholesterol and sphingolipids are also of great importance for brain function influencing cell trafficking and acting as second messenger molecules in signal transduction. On the other hand, western diets that are rich in saturated fats, have pro-inflammatory properties (Christ et al., 2019). Saturated and trans-fatty acids, and n-6 PUFAs mimic the actions of LPS-inducing pro-inflammatory processes which compromise intestinal barrier integrity as demonstrated in studies in mice (Guo et al., 2013; Williams et al., 2013).

These pro- and anti-inflammatory diets might indirectly influence neuroinflammation *via* the intestine, microbiome, and vasculature.

### The host metabolism and dietary lipids in inflammation

The effects of dietary lipids on animal physiology are heavily regulated at the cellular level by individual metabolism. Cellular organelles, such as mitochondria and peroxisomes, play central roles in dietary lipid catabolism/anabolism and productions of lipid mediators vital for signaling and metabolism. These organelles influence the impact that dietary lipids have on animal physiology. Very long-chain fatty acids (VLCFAs) derived from vegetable oils, nuts, and seeds (Tenreiro et al., 2013) are normally catabolized by peroxisomes and can be lipotoxic to any tissue if not metabolized. Accumulation of VLCFAs is linked to neurodegeneration and has been found in AD brains along with reduced peroxisome activity (Kou et al., 2011). A study carried out in *D. melanogaster* (Di Cara et al., 2018), found that peroxisomal dysfunction specifically in the intestinal epithelial cells damages intestinal epithelial integrity resulting in dysplasia, and promotes the systemic accumulation of non-esterified fatty acids. These fatty acids trigger intestinal inflammation and decrease animal survival, confirming that abnormal lipid metabolism affects inflammation. Likewise, the oxidation of long-chain fatty acids by mitochondria is protective, as mitochondria defects lead to an accumulation of long chain fatty acids in the gut epithelium which induces high levels of oxidative stress (Swanson et al., 2012; Custers et al., 2022) and inflammation that affects ENS neurotransmission (Chen et al., 2019).

The brain has the highest cholesterol content in the body as the main component of axonal myelin sheets, essential to maintain the physiological functions of the brain such as cognition and movement (Rhea and Banks, 2021). An excess of cholesterol can be damaging and therefore needs to be oxidated into the oxysterol 24S-OH-Chol by the neuronal enzyme CYP46A1. 24S-OH-Chol is then released into the circulation by neurons and eliminated. As oxysterols can diffuse through plasma membranes, they can then cross the BBB. AD patients show a reduction in circulating levels of 24S-OH-Chol, because of decreased expression of the CYP46 enzyme in neuronal cells. Oxysterols are also present in plasma mainly in the form of 27-OH-Chol as a product of the mitochondrial CYP27A1 enzyme activity in the liver. Recent works suggested that 27-OH-Chol accumulation triggers AD-related pathological changes. Rabbits fed a cholesterol-rich diet showed increased serum cholesterol, increased 27-OH-Chol in the hippocampus, and pathological hallmarks similar to those found in AD, such as microglia activation, brain atrophy, and cognitive deficits (Brooks et al., 2017). Therefore, it is suggested that dietary cholesterol could induce neurodegeneration if there is excessive production of 27-OH-Chol by the mitochondria in the liver.

### Diet and microbiota-derived metabolites in NDs

Important lipids for most physiological functions are not just dietary derived but are also products of gut microbiota metabolism. In mice and humans, it has been shown that the gut microbiota can affect lipid metabolism and lipid levels in the blood and different tissues (Chatelier et al., 2013; Cotillard et al., 2013; Lam et al., 2015; Just et al., 2018). However, dietary lipids can also alter the gut microbiota composition either by acting as substrates for bacterial metabolic processes or by diminishing bacterial growth due to their toxicity towards certain bacterial species (e.g. *Bacteroides, Clostridium,* and *Roseburia*) (Agans et al., 2018; Schoeler and Caesar, 2019). As a result, dietary lipids can shape their own metabolism by altering the balance of enteric bacteria that are able to metabolize them. SCFAs in particular are the metabolic end-products of gut-commensal fermentation of fibrous food, that have neuroprotective roles (Onyango et al., 2015; Dinan and Cryan, 2017; Baxter et al., 2019; Custers et al., 2022). For example, in a study conducted by (Kong et al., 2018), 16S rRNA gene sequencing and gas chromatography-mass spectrometry analyses in a *Drosophila* model of AD, showed decrease of *Lactobacillus* and *Acetobacter* species that correlate to a dramatic decrease of SCFA acetate, which is the most abundant SCFA. Concurrently, in *Drosophila* models of PD, the administration of sodium-butyrate reduces degeneration of dopaminergic neurons and improves the locomotor defects in a pan-neuronal transgenic fly model expressing mutant-human-α-Synuclein (St Laurent et al., 2013). SCFAs have also been linked to the maintenance of gut and immune homeostasis in mammalian systems, demonstrating a neuro-immunoendocrine regulatory role in the brain (Silva et al., 2020; Chen et al., 2022). For example, dietary butyrate has an anti-inflammatory effect in the gut (Baxter et al., 2019) and in the brain by influencing BBB permeability (Chen et al., 2022).

#### Vitamins and NDs

Other nutrients such as vitamins also influence gut and brain health through their antioxidant activities (Qi et al., 2019; Hung et al., 2020; Komleva et al., 2021). Dietary vitamin group B, such as B3 (niacin), B9 (folic acid), and vitamin B12 (cobalamin) effect learning, memory, and overall cognition (Virmani et al., 2013; Staff and Windebank, 2014). Vitamin B3 (niacin) improves locomotor deficits of PD patients and PD *Drosophila* models (Jia et al., 2008; Wakade et al., 2021). In a double blind-study conducted by (Wakade et al., 2021), 47 patients were given either a placebo or niacin supplements. After 12 months of daily supplementation and clinical tests assessing motor and non-motor quality of life symptoms and biochemical tests assessing inflammatory cytokine profiles, 42 patients exhibited an improvement in motor, and cognitive symptoms. Additionally, while the levels of many inflammatory cytokines did not change, anti-inflammatory cytokine IL10 was upregulated. Other essential vitamins of group of B do not derive from the diet but are supplied by the microbiota (Morowitz et al., 2011; Virmani et al., 2013). Therefore, dietary metabolites, not just lipids, may have neuroprotective activities within the DMGB axis to prevent the onset of NDs.

## The diet-microbiota-gut-brain axis: The gut microbiota

Over 100 years ago Elie Metchnikoff, Nobel Prize laureate for his discovery of the macrophage and phagocytosis (Dinan and Cryan, 2015), dedicated the last years of his career to the study of longevity. He proposed that people in Eastern Europe lived longer due to the high amounts of fermented foods they ate, which contained lactic acid bacteria. Later studies in germ-free mice demonstrated that germ-free animals lived longer than controls (Gustafsson 1946), suggesting that a direct link exists between microbiota, microbiota-derived metabolites, and aging. It is now well established that the microbiota and brain can communicate through different routes.

### Microbiota-brain communication *via* DMV

One line of communication between the gut microbiota and the central brain occurs *via* the DMV as shown by studies in mice demonstrating the anxiolytic effect of intestinal *Bifidobacterium longum* on animals that were subject to vagotomy (Bercik et al., 2011). As discussed above, it has also been proposed that commensal bacteria can secrete prion-like peptides that could trigger other proteins in the gut to aggregate (Chen et al., 2016; Chen et al., 2019). These misfolded aggregated proteins then spread through the intestine and the ENS to the brain. Protein aggregation of NDs peptides reach the brain *via* the DMV and a prion-like mechanism (Braak et al., 2003). This hypothesis was confirmed from preliminary data in *Rattus norvegicus* fed with bacteria that produce the prion-like protein, curli, which resulted in α-Synuclein deposition accumulation in the gut and brain (Chen et al., 2016).

#### Microbiota as a source of neurotransmitters

The gut microbiota also communicates with the brain by altering the level of neurotransmitter precursors. For example, high concentrations of *Bifidobacterium infantis* have been shown to increase plasma tryptophan levels and thus influence central 5-hydroxytryptamine transmission (Desbonnet et al., 2008; O’Mahony et al., 2015). Furthermore, commensal bacteria can synthesize and release neurotransmitters, such as the inhibitory neurotransmitter γ-aminobutyric acid (GABA), which can be produced by *Lactobacillus* and *Bifidobacterium* species. Likewise, *Candida*, *Streptococcus*, *Escherichia*, and *Enterococcus* species have been reported to produce serotonin, *Bacillus* can produce dopamine, and certain *Lactobacillus* species can produce acetylcholine (Lyte, 2013; 2014). These microbially synthesized neurotransmitters cross the mucosal layer of the intestines and mediate physiological events in the brain. Likewise, SCFAs such as propionate, butyrate, and acetate, are also important metabolic products of gut microbial activity. These SCFAs can affect the brain indirectly or directly either by acting as ligands for G-protein coupled receptors or in the case of butyrate as epigenetic modulators of histone deacetylases to control transcriptional changes that affect neuronal functions (Galland, 2014; Stilling et al., 2014; Canfora et al., 2015; Paul et al., 2015; Stilling et al., 2016).

### Microbiota composition and NDs

In its abundance, the human microbiome is composed of around 1.3 more cells than cells of the human body (>200 taxa) (Sender et al., 2016; Yang et al., 2020), acting like a major organ system that contributes to physiological health. In fact, dysbiosis of gut commensal populations leads to metabolic and inflammatory diseases (Dinan and Cryan, 2017). Neurological diseases, such as NDs are co-morbid with gastrointestinal alterations (Vandvik et al., 2004; Sampson and Sarkis, 2015). It has indeed been reported that in both mouse and *Drosophila* models of human PD, the neurodegenerative pathologies in the brain have been observed in concurrence with commensal dysbiosis in the gut (Erny et al., 2015; Challis et al., 2020; Peterson, 2020; Kitani-Morii et al., 2021; Rydbom et al., 2021).

At a phylum level, the microbiome is primarily defined by two dominant bacterial phylotypes, *Bacteroidetes* and *Firmicutes* with *Proteobacteria, Actinobacteria, Fusobacteria* and *Verrucomicrobia* present in relatively low abundance (Qin et al., 2010; Lankelma et al., 2015). In humans, mice, and *Drosophila*, the phyla *Firmicutes* dominate the gut, where the *Firmicutes* is composed of many *Lactobacillus* species (Newell and Douglas, 2014; Trinder et al., 2017; Douglas, 2018). These *Lactobacillus* species have been found to influence host metabolism and behavioral pathologies of NDs in various gut-commensal studies in mammals and *Drosophila* (Newell and Douglas, 2014). For example, *L. plantarum* in mammals influences gut-lipid metabolism, increasing butyrate which has anti-inflammatory effects (Storelli et al., 2011). In *Drosophila, Lactobacillus plantarum* also alters gut metabolism such as the insulin signaling pathway implicated in the pathogenesis of NDs (Storelli et al., 2011; Newell and Douglas, 2014; Spinelli et al., 2019; Komleva et al., 2021). Moreover, *L. plantarum* also ameliorates age-dependent memory impairment in the mouse (Castelli et al., 2021) and *Drosophila* models of NDs (Schretter et al., 2018; Schretter, 2020). Studies in *Drosophila* have identified *Lactobacillus brevis* as the culprit behind the locomotion deficit of PD pathogenesis in a *Drosophila* PD model (Schretter et al., 2018). Therefore, in humans, interaction of *Lactobacillus*, or other undiscovered or unrecognized species that influence the gut-brain axis may hold the key to unlocking the aetiologies of NDs. However, sequencing and understanding the human microbiome is much more complex than a mouse or a fly.

In humans, the gut microbiota has recently been sequenced in patients with PD in a study carried out by (Scheperjans et al., 2015). They performed metagenomic analyses of 72 PD patients and 72 matched controls. The results indicated a major reduction in the levels of Prevotellaceae in the patients and a positive association between the levels of Enterobacteriaceae and the severity of postural instability and gait difficulty. However, the authors did not claim either the temporal or causal relationship between the gut microbiota and the features of the disease. Another analysis of microbiota composition in PD pointed to a reduction in butyrate-producing bacteria in feces and *Faecalibacterium* in the mucosa of PD (Keshavarzian et al., 2015).

Despite all the encouraging studies pointing to the microbiota as a potential therapeutic target for PD, to date, there is no conclusive evidence as of yet showing that microbiota transplants can ameliorate PD pathology. More research is needed to determine the relative role of the microbiome in PD.

A detailed analysis of the microbiota in AD patients is lacking, preliminary data published in preprint format has implicated the microbiota in the accumulation of amyloid plaques in a mouse model of AD (Harach et al., 2015). In this study, the authors generated a transgenic AD mouse model under germ-free conditions and found a dramatic reduction of cerebral Aβ amyloid pathology when compared to AD animals, which had normal intestinal microbiota. Colonization of germ-free AD mice with microbiota harvested from conventionally raised AD mice dramatically increased cerebral Aβ pathology. This was similarly observed in another study showing that antibiotic treatment could limit Aβ pathology and neuroinflammation (Minter et al., 2016). These data offer hope for the future generation of a novel microbiota-based approach to ameliorate symptoms of AD.

Gut-brain communication also occurs *via* the immune system. Signaling from gut epithelial cell-secreted peptides, bacterial-derived molecules, and cytokines can activate gut resident immune cells to secrete gradients of cytokines that pass the BBB and communicate with cells in the brain. The activation of microglia, the brain’s resident immune cells, plays a central role in neuroinflammatory processes in aging and NDs (Jyothi et al., 2015). The activation of microglia appears to be influenced by the gut microbiome (Erny et al., 2015). This data suggested that neuroinflammation can be regulated by targeting the gut microbiome using probiotic approaches.

In conclusion, the microbiota-gut-brain axis is becoming increasingly recognized in the context of NDs. However, further research is needed to define how the microbes communicate with the host and to understand the interactions between the diet, the microbiota and the intestinal epithelium.

## The diet-microbiota-gut-brain axis: The gut

### The GI tract and ENS interactions

The GI tract is a very complex organ with essential functions such as absorbing and processing of ingested food to fulfill the energy demands of development, reproduction, and survival (Woods et al., 1998). The GI tract is also a major source of neuronal and endocrine signals, which modulate food intake and the activity of other organs, such as the pancreas and the brain (Kabouridis and Pachnis, 2015; Abot et al., 2018). Additionally, the GI tract forms the largest and most important immune epithelial barrier against external dangers posed by ingested pathogens. Under ideal circumstances, the GI tract maintains a mutualistic and symbiotic relationship between a diverse and dynamic community of microorganisms and the host (Peterson and Artis, 2014; Abot et al., 2018). Here we will review the mechanisms by which the gut and gut-microbiota interactions might influence the brain activities in health and in the context of NDs while we refer the readers to a review by (Richards et al., 2021) for a detailed description of the mechanisms of gut-brain communications. The GI tract is also host to the ENS, a highly dense neuronal network that acts independently of the CNS (Fleming et al., 2020). The ENS presents a similar organization to the CNS brain regarding structure, function and pluri-chemical transmission. ENS neurons modulate a range of physiological activity, such as gut motility, intestinal permeability, intestinal immunity, enteric reflex, and enteroendocrine signaling (Carabotti et al., 2015), therefore playing a role in local inflammation (Fleming et al., 2020). The ENS communicates with the CNS *via* three primary neuronal types, intrinsic primary afferent neurons, motor neurons, and interneurons (Fleming et al., 2020). Communication between the neurons of the ENS and the PNS/CNS are carried out by compounds like acetylcholine, and reactive nitrogen species like Nitric oxide (Fleming et al., 2020). The primary method of communication between the ENS and CNS is facilitated by the bidirectional, 10th cranial nerve, the DMV in mammals and *Drosophila* (Breit et al., 2018).

The DMV is the main contributor of the parasympathetic nervous system. Exiting the medulla oblongata, the DMV delivers impulses from the brain to innervate peristalsis/digestion during a parasympathetic relaxed state. In return, the intestinal epithelium delivers signals to the brain communicating the state of the gut through the ENS *via* the DMV. The efferent vagal nerves regulate gastrointestinal secretory and motor function, and also the activity in the endocrine system of the gut, while the vagal afferents allow gut-brain information flow from the gut to the CNS. Vagal afferent signaling has been implicated modulating mood and affect, including distinct forms of anxiety and fear and are involved in the activation/regulation of the Hypothalamic Pituitary Adreno axis, which coordinates the adaptive responses to stressors of any kind (Tsigos and Chrousos, 2002; Howland, 2014). The DMV also has an immunomodulatory role as the cholinergic anti-inflammatory pathway (Goverse et al., 2016; Breit et al., 2018), making the DMV a major component of the neuroendocrine-immune axis. The appearance of pathogens in the gut activates resident innate immune cells to release cytokines. These in turn activate sensory fibers that ascend the DMV. Increasing efferent signals in the DMV suppresses peripheral cytokine release through macrophage nicotinic receptors and the cholinergic anti-inflammatory pathway. Thus, experimental activation of the cholinergic anti-inflammatory pathway by direct electrical stimulation of the efferent DMV was found to inhibit the synthesis of TNF-α in the liver, spleen, and heart, and lowers serum concentrations of TNF-α (Borovikova et al., 2000; Bernik et al., 2002). This regulation of the local and systemic inflammatory signals is essential to reduce metaflammation linked to neuroinflammation.

### Humoral factors of the gut-brain communication

Gut communication to the brain does not solely rely on neurotransmission through the DMV but it can also be humoral through the secretion of metabolites processed from the diet by the microbiota or the gut epithelium. Metabolites can have neuroprotective trophic action (Liu, 2018) promoting neuron survival and plasticity in both the ENS and the CNS (Figure 1) (Liu et al., 2019). In a study conducted by (Vidal-Martínez et al., 2016) and colleagues, transgenic mice overexpressing mutant human α-synuclein developed PD-like enteric neuropathology. The animals accumulated α-synuclein aggregates in the intestinal epithelium and ENS nerves and exhibited gut motility problems that result in constipation. Treatments with FTY720, a sphingosine analog rescued the defects in the α-synuclein transgenic mice. FTY720 mediated the accumulation of the brain-derived neurotrophic factor that activates TrkB receptors in the gut reducing ENS synucleinopathy and improving gut motility (Vidal-Martinez et al., 2016).

The ENS and the intestinal epithelial cells also release peptides that can have local or distal effects. The battery of neuropeptides released in the intestines can affect the immune system or the CNS to control inflammatory responses, hormonal secretion, and metabolic activities. Gut peptides are mainly secreted from enteroendocrine cells in the GI tract. Neuropeptides and peptide hormones are released mainly by cell of the CNS and ENS executing in response to stress, metabolic, and commensals signaling. The Neuropeptide Y (NPY) is involved in controlling inflammation, pain, emotion, mood, cognition, stress responses, and metabolism. In the gut, NPY is expressed mainly by the ENS, where it regulates enteric inflammation. This is demonstrated by the observation that NPY-containing nerve fibers of the ENS are in close contact with immune cells in the mouse ileum lamina propria (Shibata et al., 2008). Specifically, NPY promotes colonic inflammation. A growing body of literature, both in humans and rodent models, suggests that brain NPY levels are altered in some neurodegenerative and neuroimmune diseases. NPY stimulates neuronal survival and neuroproliferation, attenuates neuroinflammation, and counteracts depressive symptoms and weight loss present in NDs. Ultimately, the extent to which these enteric peptides expression/repression in the gut is linked to NDs pathogenesis has not been established yet and the topic requires further investigation.

### The immune system in the GI tract heavily influences gut-brain communication

The intestinal immune cells constantly face a large number of antigens that are obtained from either food or the intestinal microbiota. Intestinal CD4^+^ T helper cells are highly involved in mucosal immunity. Enteric glial cells are essential for the integrity of the bowel. The presence of different bacterial species determines the pro- or anti-inflammatory state of CD4^+^ T-cells (Ivanov et al., 2009), which highlights the importance of the intestinal microbiota in the preservation of mucosal homeostasis. Failure to inhibit pro-inflammatory immune responses increases intestinal inflammation and may contribute to the development of immune-mediated inflammatory diseases. A loss of enteric glial cells also leads to severe inflammation of the intestines. The increased inflammation of the gut affects the permeability of the intestinal barrier activating resident immune cells and leading to the accumulation of inflammatory mediators and microbial-derived metabolites such as LPS in the circulatory system triggering systemic inflammation and metabolic dysfunction (Lancaster et al., 2018). Humoral pathways of gut inflammation spreading to the brain can damage the BBB. Multiple reports have shown that the BBB is damaged in PD patients (Pisani et al., 2012) and LPS (Gray and Woulfe, 2015) induced PD animal models exhibited disrupted BBB (Varatharaj and Galea, 2017). If the BBB is damaged, proinflammatory cytokines and immune cells such as T-cells (Engelhardt, 2017) and mast cells (Jones et al., 2019) from peripheral inflammation are able to enter the brain. These series of events are now considered the current model mechanism of disease of NDs that could be applied to familiar and sporadic forms of NDs and support a scenario that points at peripheral inflammation and alteration of the DMGB as main triggers of pathogenesis.

### Metabolic alteration in the GI as markers of NDs

Metabolic alteration and inflammation of the intestine are two features observed in the most common NDs. How metabolic alteration occurs and what pathway is principally involved in the intestinal epithelium when the diseases seed, is not understood. Damage or functional alteration of essential organelles such as mitochondria and peroxisomes, have been found to accompany different pathologies of disease. Mitochondria and peroxisomes are highly metabolic cellular organelles, ubiquitously conserved across eukaryotes, from yeast to humans (Osellame et al., 2012; Smith and Aitchison, 2013; Baron et al., 2016). Both organelles participate in a broad range of conserved cellular-metabolic processes, most notably β-oxidation of medium-chain, long chain and very long-chain fatty acids, the anabolism and catabolism of complex signaling lipids such as phospholipids, and synthesis and turnover of reactive oxygen and reactive nitrogen species. These metabolites are essential for supporting cellular energetic processes, limiting redox stress, mitigating inflammation, and maintaining cell structure and signaling (Osellame et al., 2012; López-Armada et al., 2013; Smith and Aitchison, 2013; Di Cara, 2020). As mentioned previously, α-Synuclein inclusion bodies have been identified in the gut and enteric neurons of PD patients prior to the onset of disease, as well as in cases of inflammatory diseases that compromise the gut epithelial barrier (Hawkes et al., 2010; Challis et al., 2020; Derkinderen et al., 2021). α-Synuclein inclusions damage cellular mitochondrial functions, therefore, linking the diseases to metabolic and signaling dysfunction of mitochondria. However other studies reported that mitochondrial damage occurs before α-Synuclein inclusions appear.

Peroxisome dysfunction has not been directly linked to the onset of NDs. Cumulative evidence has reported that peroxisome dysfunction in the intestinal epithelium causes intestinal inflammation and dysplasia as reported in *Drosophila* gut-specific peroxisomal knock-down models by (Di Cara et al., 2018). In the same study, peroxisomes were also found to modulate the gut microbiota which, as discussed herein, changes in gut microbiota are tightly connected to inflammatory diseases including NDs. Clinical reports described that peroxisome numbers and metabolic activities are lower within neurons of the post-mortem brains of patients affected by NDs such as AD (Kou et al., 2011; Cipolla and Lodhi, 2017). In particular, post-mortem brains of AD patients have high VLCFAs (substrates of peroxisome β-oxidation) and in a parallel a decrease in ether-phospholipids (products of peroxisomal metabolism) (Kou et al., 2011).

Despite these correlations, more studies are needed to establish whether alteration of mitochondria and peroxisomes in the intestinal cells could be the culprit of the metabolic dysfunction that triggers the cascade to NDs.

## Discussion

For decades the study of genetic-NDs have generated several theories on sporadic disease pathogenesis (Garden and La Spada, 2012) based on how the disease pathologically presents in the brain showing mitochondrial dysfunction, oxidative stress, aberrant neurotransmission and neuronal death. However, emerging evidence has proven that the pathologies characterized in the CNS might be a consequence of metabolic and inflammatory dysfunctions occurring in distal organs such as the gastrointestinal tract. As discussed in this review, β-amyloid plaques and α-Synuclein Lewy body inclusions that represent signs of disease in the brain of AD and PD respectively, have also been identified in enteric neurons (Barbut et al., 2019; Challis et al., 2020). These findings highlight how NDs are not confined to the brain as previously believed, and might be a product of bidirectional communication of the body with the brain *via* inter-organ communication/coordination to maintain tissue homeostasis (Peters, 2006; McHugh and Gil, 2018). Nutritional, neuronal, and inflammatory signals have been linked to NDs, thus, NDs should be studied in a multi-factorial way, where commensal, and intestinal metabolic signals that shape physiological processes should also be considered. Additionally, alteration of cellular organelles such as mitochondria and peroxisomes in the intestinal epithelium could be the culprit of the metabolic dysfunction that triggers aberrant DMGB axis communication, and in turn NDs. Damage to these organelles and their metabolic processes could occur years before the disease appears in people that develop PD or AD. Therefore, organelle dysfunction could be used as an early marker to measure DMGB axis activity as a predictor of disease. It remains to be investigated whether alteration of organelle metabolism initiates the process of metaflammation and therefore drives the cascade that leads to neuronal death and the accumulation of Aβ aggregates or α-Synuclein Lewis inclusion bodies.

Another open question that remains about the mechanisms of diseases of NDs, is whether the early distal signaling that leads to ND seeding in the brain comes exclusively from the diet-gut-brain axis or if this axis integrates metabolic and inflammatory signaling from other organs as well. For instance, the immune system has an integral part in triggering the oxidative stress and inflammation that contributes to neuronal death. Moreover, recent work identified that the liver as another integral contributor to this axis. This was specifically demonstrated in mice in which the hepatocyte-restricted expression of the human mutant variant of APP displayed peripheral metabolism of Aβ peptides and associated neurovascular inflammation, CNS neurodegeneration, and memory impairment. These animals exhibited an accumulation of triglyceride rich lipoprotein-Aβ in the circulation that might increase the permeability of the BBB, as well as the accumulation of cerebral neutral lipids and widespread aggregates and neuronal loss. This work suggests that hepatic metabolism of Aβ can cause neuroinflammation and AD. Thus, the question stands as to whether metabolic disturbances in one or multiple peripheral organs might happen in a defined sequence that ultimately consist in a disease risk factor. Dissecting these networks is a complex task and requires that all or at least many of these potential contributing factors are individually controlled. The establishment of *Drosophila* model organisms to study NDs will help to dissect the complex network of the DMGB axis and to identify whether multiple organs influence this DMGB axis in the etiology of NDs.

These new approaches that will help us investigate the origin of NDs outside the brain have expansive promise to identify early markers of disease or develop treatments capable of targeting novel molecular networks for disease prevention.

## Abbreviations

APP: Amyloid Precursor Protein; BBB: Blood Brain Barrier; BDNF: Brain Derived Neurotropic Factor; DMGB axis: Diet-Microbiota-Gut-Brain axis; DMV: Motor nucleus of the Vagus nerve; ICR: Institute of Cancer Research; NFTs: Neurofibrillary Tangles; PFFs: Pre-Formed Fibrils; SCFAs: Short Chain Fatty Acids; VLCFAs: Very Long Chain Fatty acids.
